# *Chrysanthemi Flos* extract alleviated acetaminophen-induced rat liver injury via inhibiting oxidative stress and apoptosis based on network pharmacology analysis

**DOI:** 10.1080/13880209.2021.1986077

**Published:** 2021-10-09

**Authors:** Yunfeng Zhou, Chunli Wang, Jiejian Kou, Minghui Wang, Xuli Rong, Xiaohui Pu, Xinmei Xie, Guang Han, Xiaobin Pang

**Affiliations:** a Pharmaceutical Institute, School of Pharmacy, Henan University, Kaifeng, China; b Kaifeng Key Lab for Application of Local Dendranthema morifolium in Food & Drug, Kaifeng, China; c Institutes of Traditional Chinese Medicine, Henan University, Kaifeng, China

**Keywords:** *Chrysanthemum morifolium*, drug-induced liver injury, antioxidant, anti-apoptosis

## Abstract

**Context:**

Acetaminophen (APAP) overdose is the leading cause of drug-induced liver injury. *Bianliang ziyu*, a variety of *Chrysanthemum morifolium* Ramat. (Asteraceae), has potential hepatoprotective effect. However, the mechanism is not clear yet.

**Objective:**

To investigate the hepatoprotective activity and mechanism of *Bianliang ziyu* flower ethanol extract (BZE) on APAP-induced rats based on network pharmacology.

**Materials and methods:**

Potential pathways of BZE were predicted by network pharmacology. Male Sprague-Dawley rats were pre-treated with BZE (110, 220 and 440 mg/kg, i.g.) for eight days, and then APAP (800 mg/kg, i.g.) was used to induce liver injury. After 24 h, serum and liver were collected for biochemical detection and western blot measurement.

**Results:**

Network pharmacology indicated that liver-protective effect of BZE was associated with its antioxidant and anti-apoptotic efficacy. APAP-induced liver pathological change was alleviated, and elevated serum AST and ALT were reduced by BZE (440 mg/kg) (from 66.45 to 22.64 U/L and from 59.59 to 17.49 U/L, respectively). BZE (440 mg/kg) reduced the ROS to 65.50%, and upregulated SOD and GSH by 212.92% and 175.38%, respectively. In addition, BZE (440 mg/kg) increased levels of p-AMPK, p-GSK3β, HO-1 and NQO1, ranging from 1.66- to 10.29-fold compared to APAP group, and promoted nuclear translocation of Nrf2. BZE also inhibited apoptosis induced by APAP through the PI3K–Akt pathway and restored the ability of mitochondrial biogenesis.

**Discussion and conclusions:**

Our study demonstrated that BZE protected rats from APAP-induced liver injury through antioxidant and anti-apoptotic pathways, suggesting BZE could be further developed as a potential liver-protecting agent.

## Introduction

The liver is a vital organ for drug metabolism and detoxification with high sensitivity to some drugs or their metabolites, which can lead to hepatic injury (Singh et al. 2015; Rada et al. 2018). Acetaminophen (*n*-acetyl-aminophenol, APAP), also known as paracetamol, is the most commonly used antipyretic and analgesic drug, mainly used for fever, migraine, neuralgia, joint pain, etc. (Zhang et al. 2016). For more than half a century, people benefitted from the application of APAP to fight diseases and also suffered adverse effects caused by APAP. The most serious adverse reaction of APAP is hepatotoxicity resulting from overdose or long-term use, which can lead to the necrosis of liver lobule centre or severe liver failure (Harris and Myers 1997). Liver toxicity induced by APAP overdose is the most common cause of acute liver failure in the United States and other western countries (Yan et al. 2018).

Increasing attention has been paid to the application of natural plants for the prevention and treatment of modern diseases due to their safety and multiple effects. *Chrysanthemi Flos* (*C. Flos*) known as ‘Ju Hua’ in China, derived from the dry flowers of *Chrysanthemum morifolium* Ramat. (Asteraceae), is one of the most frequently used traditional Chinese herb medicines with the function of dispelling wind, dissipating heat, clearing the liver and improving eyesight (Mokaddem-Daroui et al. 2012), and is also used as a health-care edible herb medicine in China. The antimicrobial, antioxidant, cardiovascular protection, anticancer and anti-inflammatory effects of *C. Flos* have been widely reported (Yuan et al. 2020). *Bianliang ziyu* is one of the most famous varieties of *Chrysanthemum morifolium* in Kaifeng, China. Currently, the flower of *Bianliang ziyu* is only used for ornamental value, but not for medical purpose. Our previous studies have demonstrated that the ethanol extract of *Bianliang ziyu* flower (BZE) exerted protective effects in alcohol and CCl_4_-induced liver injury models (Tian et al. 2019). However, the detailed mechanism of BZE in liver protection remains to be illustrated.

As a critical component of rapidly developed system biology, network pharmacology was proposed in 2007 and provided a new sight to reveal the mechanism of a complex system with multiple components, including traditional Chinese medicine (TCM) (Zhang R et al. 2019). The bioactive compounds of single herb or TCM formula could be identified and the potential targets of these compounds could be collected based on network pharmacology through many online databases. By constructing multiple network models, such as compound-target network and protein–protein interaction (PPI) network, we could obtain the core targets and crucial pathways, and further elucidate the molecular mechanism of TCM (Lin et al. 2020). Network pharmacology has successfully applied to uncover the potential mechanism of many complex systems in TCM (Tian G et al. 2019; Wang et al. 2019).

In this research, network pharmacology analysis was adopted to predict the putative targets and pathways of the bioactive compounds in BZE against drug-induced liver injury first. Then, a rat model of liver injury induced by APAP was established to verify the hepatoprotective mechanism of BZE provided by network pharmacology.

## Materials and methods

### Screening of major bioactive compounds and targets of *C. Flos*

The compounds of *C. Flos* were searched from TCMSP database (https://tcmspw.com/tcmsp.php, version 2.3), a comprehensive website that provided main components of common Chinese herbal medicines and parameters in absorption, distribution, metabolism and excretion (ADME) of these components. The compounds with oral bioavailability (OB) ≥30% and drug-likeness (DL) ≥0.18 were selected for further target prediction. Afterwards, the structures of these bioactive compounds were uploaded to Swiss TargetPrediction website (http://www.swisstargetprediction.ch/) (Daina et al. 2019) to obtain the potential targets. Only the targets with higher reliability (probability ≥50%) were collected for further study.

### Collection of targets related to drug-induced liver injury

According to a previous report, the key word ‘drug-induced liver injury’ was imported to DrugBank (https://go.drugbank.com/drugs), GeneCards (https://www.genecards.org/) and OMIM (https://www.omim.org/) database to acquire liver injury related targets, among of which the repetitions were removed (Huang et al. 2020).

### Construction of component-target and protein–protein interaction networks

All the targets collected above were transferred to official gene symbol by UniProt database (https://www.uniprot.org/), and the component-target network of *C. Flos* was established by Cytoscape 3.8.1 (Shannon et al. 2003). The common targets of bioactive compounds and liver injury were regarded as the putative targets of *C. Flos* against liver injury. PPI network was established by uploading these common targets to STRING website (https://string-db.org/). Species was limited to ‘*Homo sapiens*’ and the confidence score was set as more than 0.4.

### GO and KEGG enrichment analysis

Gene ontology (GO) and Kyoto Encyclopaedia of Genes and Genomes (KEGG) enrichment analysis for identified common targets were performed by DAVID database (https://david.ncifcrf.gov/). The biological progresses (BPs) in GO analysis and the pathways in KEGG analysis were considered as statistical significance based on *p* < 0.05.

### Chemicals and reagents

APAP was purchased from Aladdin Bio-Chem Technology Co., Ltd. (Shanghai, China). Kits for alanine aminotransferase (ALT), aspartate aminotransferase (AST), ROS, malondialdehyde (MDA), GSH and SOD detection were provided by Nanjing Jiancheng Bioengineering Institute (Nanjing, China). A histone extraction kit, rhodamine 123 (Rh123) and enhanced chemiluminescence kits were bought from Beyotime Biotechnology (Shanghai, China). Haematoxylin and eosin (H&E) and BCA protein quantitative kits were obtained from Solarbio Science & Technology Co., Ltd. (Beijing, China). Antibodies for p-Akt, Akt, caspase-3, Bax, Bcl-2, p-AMPK, AMPK, p-GSK-3β (Ser9), GSK-3β, Nrf2, HO-1, NRF1 and TFAM were purchased from Cell Signaling Technology (Danvers, MA); anti-NQO1 antibody was obtained from Abcam (Cambridge, MA). Rabbit anti-β-actin, anti-mouse IgG, anti-rabbit IgG, rabbit anti-PPAR-γ and mouse anti-PGC-1α antibodies were offered by Proteintech Group, Inc. (Wuhan, China). All other reagents were analytical grade.

### Preparation of BZE

The flower of *Bianliang ziyu* was collected from Kaifeng, China and was identified by associate professor Yanli Zhao from Kaifeng Agricultural Science Institute. A voucher specimen was deposited in Herbarium of School of Pharmacy, Henan University (no. 20180502). BZE was prepared in accordance with previously developed method by our laboratory (Tian Z et al. 2019). Briefly, the flower of *Bianliang ziyu* was dried and smashed, and the powder was refluxed with 40 volume of 70% ethanol at 80 °C for 2 h. After filtration, the extracting solution was concentrated by rotary evaporator. The dried extract power was stored at 4 °C and dissolved in double distilled water to appropriate concentration when used. The total flavonoid in the extract was 30.72%, determined by ultraviolet spectrophotometry.

### Animals

Male Sprague-Dawley rats (200–220 g) were purchased from the Center of Experimental Animals of Henan Province (Zhengzhou, China). Rats were kept in a controlled environment (temperature: 25 ± 2 °C; humidity: 60 ± 5%; 12 h dark/light cycle). The animals had free access to food and water. All studies were carried out in compliance with the Guidelines for the Care and Use of Laboratory Animals (Ministry of Science and Technology of the People’s Republic of China), and all animal protocols were approved by the ethics committee of Henan University.

### Experimental design

After one-week adaption, rats were randomly divided into the following five groups: control group, APAP group and APAP + BZE groups (110, 220 and 440 mg/kg) (six rats per group). The doses were chosen according to our previous studies (Tian Z et al. 2019). The rats in the APAP + BZE groups were treated with BZE for eight consecutive days (i.g.). On the ninth day, APAP (800 mg/kg, i.g.) was administered to rats 0.5 h after BZE treatment, and rats in control group were treated with equivalent volume of sodium carboxymethyl cellulose solution. After 24 h, blood of rats was collected and liver was removed and stored at −80 °C until analysis.

### Biochemical index assays

The blood was centrifuged at 3000 rpm for 10 min at 4 °C to obtain serum. Liver tissues were weighted and ground with saline solution to produce 10% tissue homogenates. The contents of ALT, AST, GSH and SOD in the serum and the levels of MDA and ROS in liver tissue were measured by corresponding kits according to manufactures’ instructions.

### Histopathological evaluation

Histopathological changes of liver were observed by H&E staining. In brief, the liver tissues were fixed in 4% paraformaldehyde for 24 h, and then were dehydrated in graded ethanol solutions. After embedded in paraffin, the liver tissues were cut into 5-μm sections. The paraffin-embedded sections were stained with H&E according to a standard protocol. The stained specimens were observed by a light microscope.

### Mitochondrial membrane potential detection

Mitochondrial membrane potential was assayed by Rh123 staining. Rh123 is a fluorescent dye that can aggregate into the mitochondria of living cell and present yellowish green fluorescence. Approximately, 0.1 g of fresh liver tissue was isolated, and the mitochondria were extracted and mitochondrial suspension was mixed with diluted Rh123. After incubation for 5 min at room temperature, the fluorescence intensity was measured by multifunctional microplate reader with 507 nm for excitation and 529 nm for emission.

### Western blot analysis

Total protein was extracted from hepatic tissue samples (0.1 g) using RIPA buffer containing a protease inhibitor cocktail and protein concentrations were measured by BCA kits. Extracted proteins were subjected to sodium dodecyl sulphate polyacrylamide gel electrophoresis (SDS-PAGE) and transferred to polyvinylidene difluoride (PVDF) membranes. Then, the PVDF membranes were blocked with 1 × TBST containing 5% skim milk for 2 h at room temperature, and the membranes were incubated with primary antibodies overnight at 4 °C. After washes for three times with TBST, the membranes were incubated with secondary antibody for 2 h. The protein band was detected by enhanced chemiluminescence plus kit and the grey value was evaluated by ImageJ2X.

### Statistical analysis

All the data were expressed as the mean ± standard deviation (SD) and were processed by SPSS 18.0 (SPSS Inc., Chicago, IL). One-way analysis of variance (ANOVA) followed by Fisher’s least significant difference (LSD) *post hoc* test was applied to compare the difference among groups. *p* < 0.05 was considered statistically significant.

## Results

### Potential targets of *C. Flos* and the construction of compound-target network

**Figure 1.**
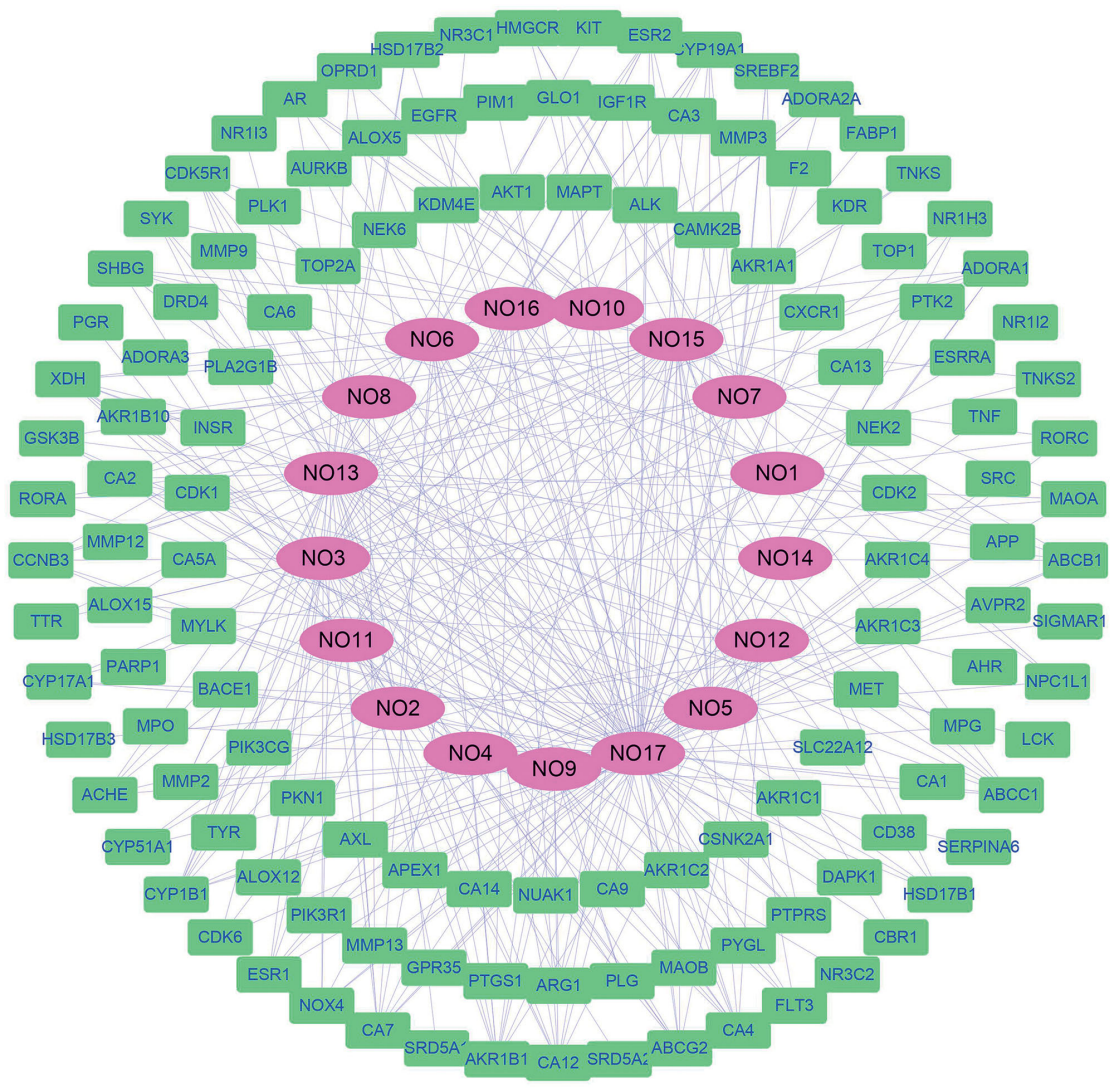
The compound-target network of *C. Flos*. The ellipse in purple indicates the bioactive compounds and the rectangle in green indicates potential targets.

A total of 359 compounds were collected in *C. Flos* from TCMSP, and 17 compounds were identified as bioactive components based on the criterion of OB ≥0.3 and DL ≥0.18, which are listed in Table 1. After the structures were imported into Swiss TargetPrediction, 139 putative targets linked to these compounds were collected. The compound-target network was visualized by Cytoscape 3.8.1 with 147 nodes and 336 edges (Figure 1(A)). Quercetin (**17**), kaempferol (**13**), luteolin (**15**) and acacetin (**3**) are top four compounds with highest degree in the network.

**Table 1.** The bioactive compounds in *Chrysanthemum Flos* collected from TCMSP database.

Compound no.	Name	MW	MF	OB (%)	DL
No. 1	(24r)-Saringosterol	428.77	C_29_H_48_O_2_	39.36	0.79
No. 2	24-Ethylcholest-4-en-3-one	412.77	C_29_H_48_O	36.08	0.76
No. 3	Acacetin	284.28	C_16_H_12_O_5_	34.97	0.24
No. 4	Artemetin	388.4	C_20_H_20_O_8_	49.55	0.48
No. 5	beta-Sitosterol	414.79	C_29_H_50_O	36.91	0.75
No. 6	Chryseriol	300.28	C_16_H_12_O_6_	35.85	0.27
No. 7	Clionasterol	414.79	C_29_H_50_O	36.91	0.75
No. 8	Cynarine	516.49	C_25_H_24_O_12_	31.76	0.68
No. 9	Diosmetin	300.28	C_16_H_12_O_6_	31.14	0.27
No. 10	Eupatorin	344.34	C_18_H_16_O_7_	30.23	0.37
No. 11	Hesperetin	302.3	C_16_H_14_O_6_	47.74	0.27
No. 12	Isorhamnetin	316.28	C_16_H_12_O_7_	49.6	0.31
No. 13	Kaempferol	286.25	C_15_H_10_O_6_	41.88	0.24
No. 14	Linarin	592.6	C_28_H_32_O_14_	39.84	0.71
No. 15	Luteolin	286.25	C_15_H_10_O_6_	36.16	0.25
No. 16	Naringenin	272.27	C_15_H_12_O_5_	59.29	0.21
No. 17	Quercetin	302.25	C_15_H_10_O_7_	46.43	0.28

MW: molecular weight; MF: molecular formula; OB: oral bioavailability; DL: drug-likeness.

### Targets related to liver injury and the PPI network of common targets

**Figure 2.**
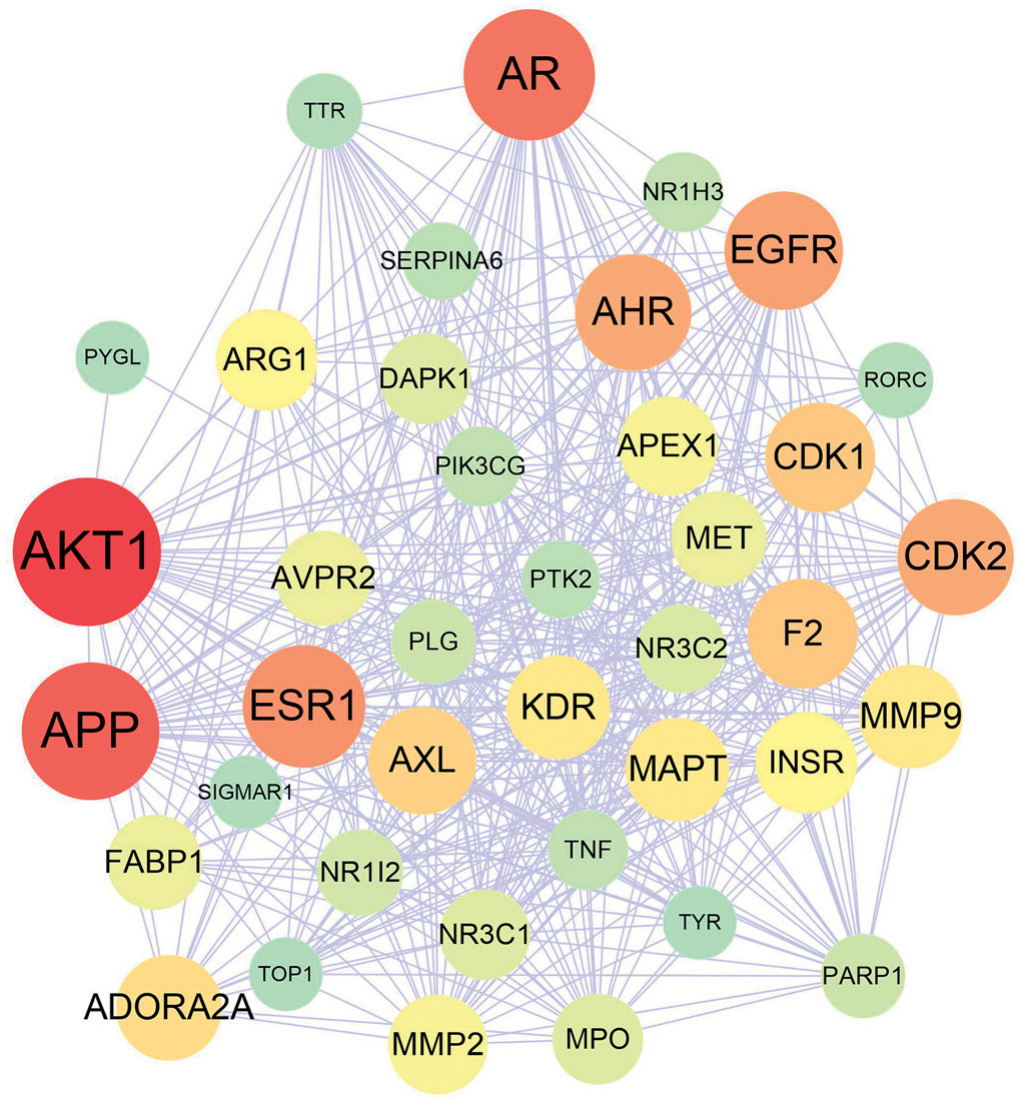
The protein–protein interaction (PPI) of the common targets. The protein with higher degree was presented with larger node and darker colour.

After searching in DrugBank, GeneCards and OMIM database and removing the repeated targets, 973 targets related to drug-induced liver injury were identified. Among of these, 39 targets were the overlapping ones with the targets of 17 bioactive compounds and were regarded as common targets. Then PPI network of 39 common targets was constructed by STRING, which had 39 nodes and 425 edges showed by Cytoscape (Figure 2), and the top five targets with highest degree were RAC-alpha serine/threonine-protein kinase (AKT1), amyloid-beta precursor protein (APP), androgen receptor (AR), oestrogen receptor (ESR1) and epidermal growth factor receptor (EGFR).

### GO and KEGG pathway enrichment analysis

DAVID database was used to performed GO and KEGG analysis to explore the potential mechanism of *C. Flos* against liver injury. The 10 main pathways in KEGG enrichment and 10 main BPs in GO analysis are exhibited in Table 2. Among them, we found that the hepatoprotective effect of *C. Flos* might be mainly involved into the positive regulation of the cell proliferation and survival through PI3K–Akt signalling pathway, negative regulation of apoptotic process and alleviate the excessive oxidative stress induced by liver injury.

**Table 2.** The main pathways and biological processes in KEGG and GO enrichment analysis.

Pathway no.	KEGG pathway	Number of gene	*p* Value	GO no.	Biological process in GO analysis	Number of gene	*p* Value
hsa04151	PI3K–Akt signalling pathway	8	6.94E-04	0007165	Signal transduction	10	9.16E-04
hsa04015	Rap1 signalling pathway	7	2.93E-04	0008284	Positive regulation of cell proliferation	9	7.79E-06
hsa04915	Oestrogen signalling pathway	6	7.04E-05	0045893	Positive regulation of transcription	9	1.61E-05
hsa04931	Insulin resistance	6	1.07E-04	0001934	Positive regulation of protein phosphorylation	8	1.24E-08
hsa04510	Focal adhesion	6	0.0021	0043066	Negative regulation of apoptotic process	8	6.24E-05
hsa04014	Ras signalling pathway	6	0.0031	0051897	Positive regulation of protein kinase B signalling	6	1.22E-06
hsa04068	FoxO signalling pathway	5	0.0029	0030522	Intracellular receptor signalling pathway	5	1.56E-06
hsa04932	Non-alcoholic fatty liver disease	5	0.0044	0070301	Cellular response to hydrogen peroxide	5	8.08E-06
hsa04370	VEGF signalling pathway	4	0.0025	0006979	Response to oxidative stress	4	0.002
hsa04012	ErbB signalling pathway	4	0.0068	1900182	Positive regulation of protein localization to nucleus	3	0.001

### Effects of BZE on serum ALT and AST levels and hepatic histopathology of APAP-treated rats

**Figure 3.**
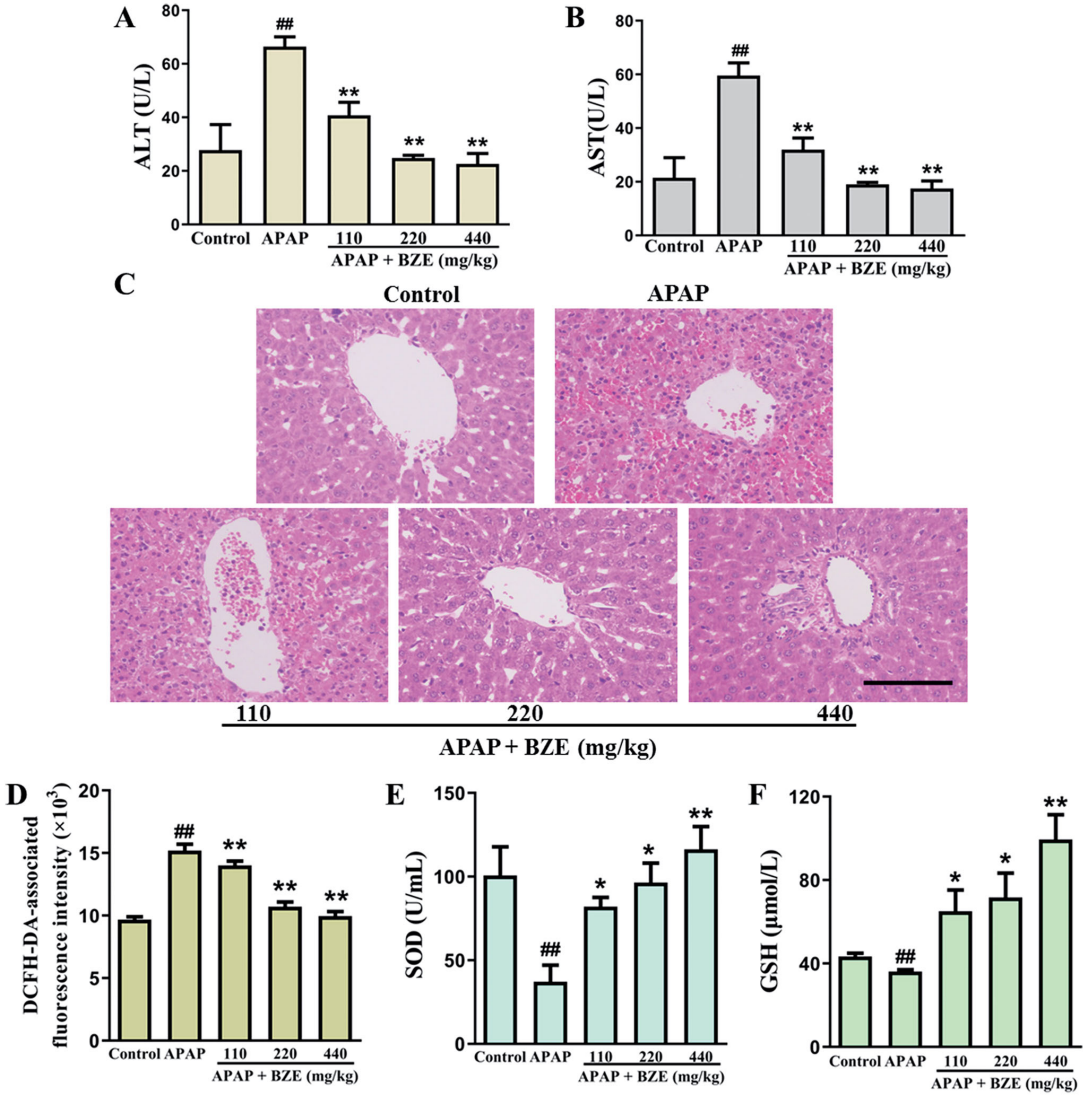
The effects of BZE on APAP-induced liver injury and oxidative stress. (A, B) The levels of ALT and AST; (C) the representative images of liver H&E staining (bar = 100 μm); (D–F) the measurement of ROS in liver, and SOD and GSH in serum of rats (means ± SD, *n* = 6); ^##^*p* < 0.01 compared to control group; *p < 0.05, **p < 0.01 compared to APAP group. APAP: acetaminophen; BZE: extract of *Bianliang ziyu* flower.

Serum ALT and AST levels are sensitive indicators of liver injury. To study the effect of BZE on liver injury caused by APAP, the levels of ALT and AST were determined. As shown in Figure 3(A,B), the levels of ALT and AST in control rats were 27.74 and 21.49 U/L, respectively. APAP administration significantly increased serum ALT and AST levels of rats (66.45 and 59.59 U/L, *p* < 0.01), which were the 2.40- and 2.77-fold compared to control group, separately. However, BZE treatment reversed abnormal increase of ALT and AST significantly (*p* < 0.01) and the concentrations of ALT and AST in serum of APAP + BZE 440 mg/kg group were approximately equal to those in control group, suggesting that BZE could remarkably alleviate hepatotoxicity caused by excessive APAP (the detailed data are displayed in Table S1).

The H&E staining of liver tissue is presented in Figure 3(C). The liver cells were orderly arranged with clear edge and abundant cytoplasm and no obvious abnormality was observed in liver sinus of control rats. APAP induced markedly pathological changes in liver tissue, presented by swollen liver cell, irregular nucleus size, hepatic cord disorder, neutrophil infiltration. BZE treatment from 110 to 440 mg/kg could reverse the abnormal changes induced by APAP with varying degrees, and the morphology and structure of liver cells in APAP + BZE 440 mg/kg group were similar to those in control group, suggesting the protective activity of BZE against APAP-induced liver injury.

### Effects of BZE on APAP-induced oxidative stress

The levels of ROS in liver tissue, and SOD and GSH in serum were measured in rats. As shown in Figure 3(D–F), APAP significantly increased the levels of ROS to 1.57-fold of control group, but remarkably decreased SOD activity and GSH content to 36.90% and 83.33% of control group (*p* < 0.01). BZE pre-treatment significantly upregulated SOD activity and GSH level in the serum and inhibited excessive ROS production in APAP-induced liver (*p* < 0.05 or *p* < 0.01), which indicated the excellent antioxidant capacity of BZE (the detailed data are displayed in Table S1).

### Effects of BZE on the AMPK/GSK3β–Nrf2 antioxidant signalling pathway

**Figure 4.**
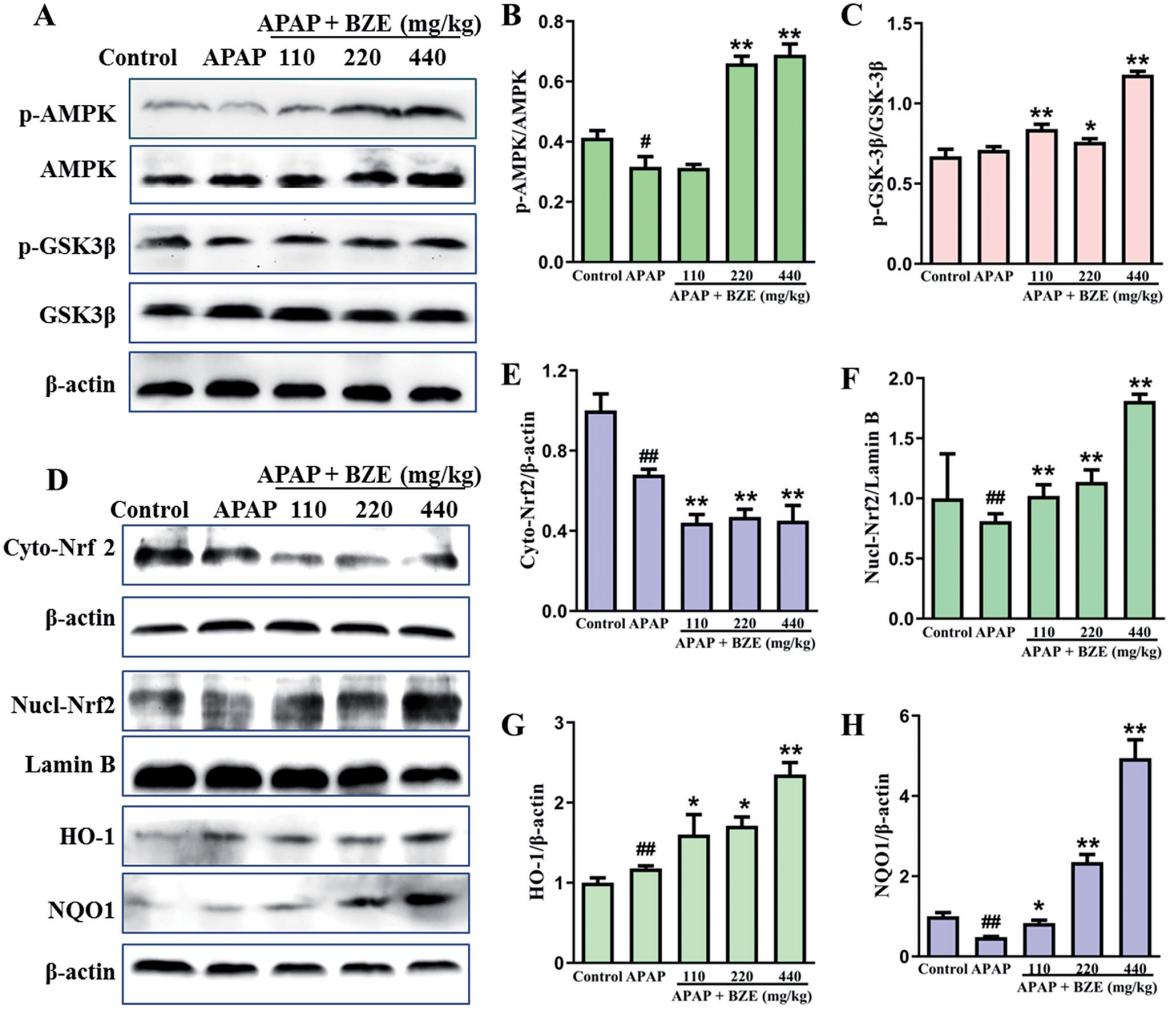
Effects of BZE on the AMPK/GSK3β–Nrf2 antioxidant signalling pathway. (A, D) The representative protein bands; (B, C and E–H) relative expression levels of p-AMPK, p-GSK3β, Nrf2 (cytoplasm), Nrf2 (nuclear), HO-1 and NQO1 (means ± SD, *n* = 3); ^#^*p* < 0.05, ^##^*p* < 0.01 compared to control group; *p < 0.05, **p < 0.01 compared to APAP group. APAP: acetaminophen; BZE: extract of *Bianliang ziyu* flower.

The antioxidant signalling mediated by AMPK/GSK3β–Nrf2 was evaluated by western blot. As shown in Figure 4, BZE significantly promoted the phosphorylation of AMPK and GSK3β (*p* < 0.05 or *p* < 0.01) and accelerated the nuclear translocation of Nrf2, displayed by remarkable decrease of Nrf2 in cytoplasm and obvious accumulation of Nrf2 in nuclear (*p* < 0.01), compared with APAP group. In addition, BZE also upregulated the protein expression of HO-1 and NQO1, the two downstream antioxidant enzymes of Nrf2, in a dose-dependent manner (*p* < 0.05 or *p* < 0.01).

### Effects of BZE on PI3K–Akt pathway and APAP-induced apoptosis

**Figure 5.**
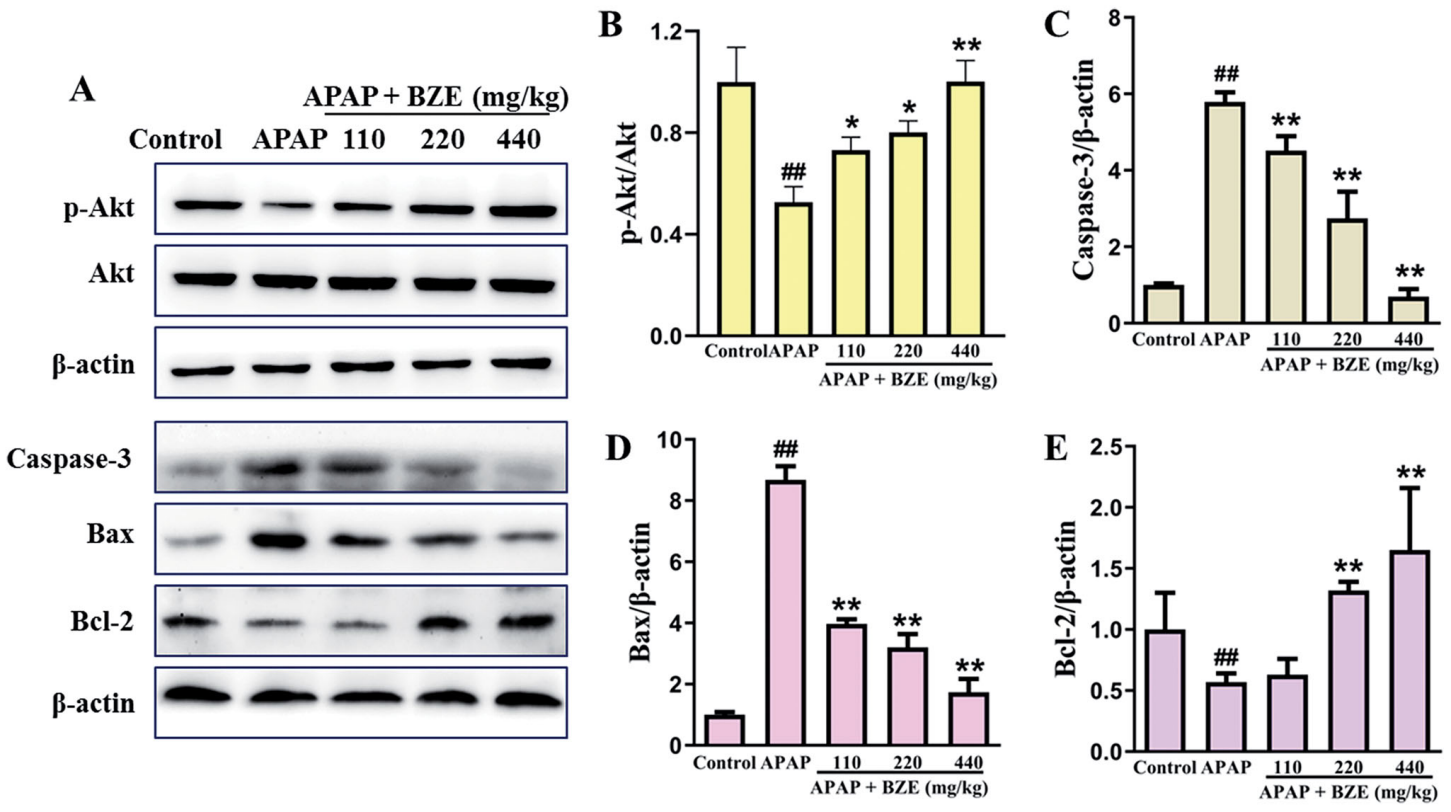
Effects of BZE on PI3K–Akt pathway and APAP-induced apoptosis. (A) Representative protein bands; (B–E) the relative expression levels of p-Akt, caspase-3, Bax and Bcl-2 (means ± SD, *n* = 6); ^##^*p* < 0.01 compared to control group; *p < 0.05, **p < 0.01 compared to APAP group. APAP: acetaminophen; BZE: extract of *Bianliang ziyu* flower.

PI3K–Akt pathway predicted by network pharmacology and the anti-apoptosis effect of BZE were detected by western blot. As shown in Figure 5, APAP significantly inhibited the phosphorylation of Akt, increased the expression of the pro-apoptotic proteins caspase-3 and Bax, and markedly reduced the level of the anti-apoptotic protein Bcl-2 (*p* < 0.01). BZE pre-treatment could remarkably upregulate the expression of p-Akt and Bcl-2 and downregulate the levels of the Bax and caspase-3 (*p* < 0.05 or *p* < 0.01). These results suggested that BZE might inhibit the APAP-induced cell apoptosis by regulating the PI3K–Akt pathway.

### Effects of BZE on mitochondrial biogenesis in liver tissues

**Figure 6.**
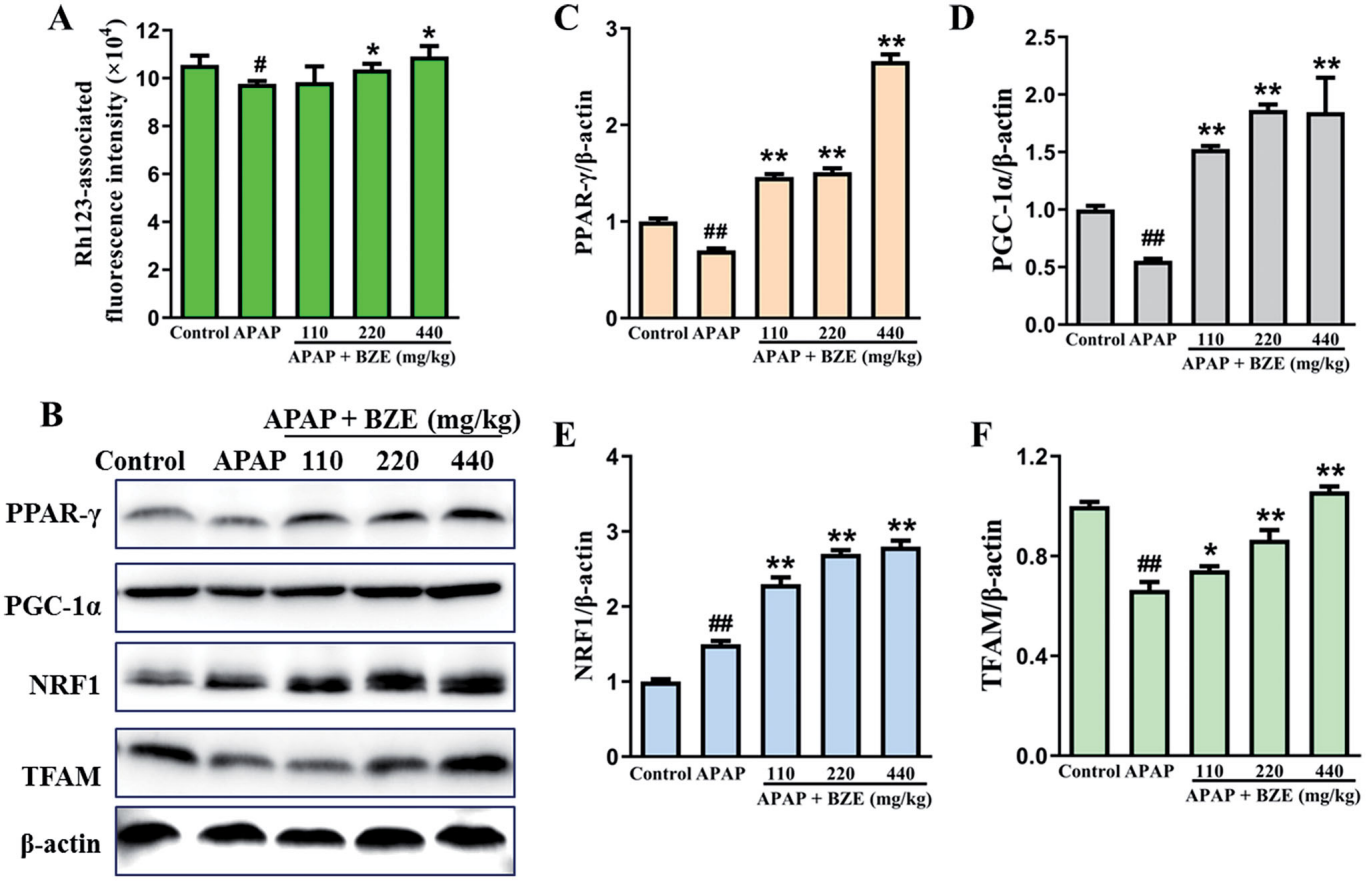
Effects of BZE on the APAP-induced mitochondrial membrane potential and mitochondrial biosynthesis-related proteins. (A) The measurement of mitochondrial membrane potential; (B) representative protein bands; (C–F) the relative expression levels of PPAR-γ, PGC-1α, NRF1 and TFAM (means ± SD, *n* = 6). ^#^*p* < 0.05, ^##^*p* < 0.01 compared to control group; *p < 0.05, **p < 0.01 compared to APAP group. APAP: acetaminophen; BZE: extract of *Bianliang ziyu* flower.

Mitochondrial membrane potential and the expression of mitochondrial biosynthesis-related proteins were detected to explore the effect of BZE on mitochondria. As shown in Figure 6, APAP caused significant reduction in mitochondrial membrane potential of liver cells, which could be significantly increased by BZE pre-treatment (*p* < 0.05), indicating that BZE could improve mitochondrial function. Moreover, BZE upregulated mitochondrial biosynthesis-related proteins, including PPAR-γ, PGC-1α, NRF1 and TFAM (*p* < 0.05 or *p* < 0.01), suggesting that BZE can improve mitochondrial function by promoting mitochondrial biogenesis.

## Discussion

Hepatotoxicity caused by APAP overdose is the main cause of liver injury events in developed countries (Rousar et al. 2012). *C. Flos* can be used both in diet and medicine, and has been confirmed to present excellent protective effects in carbon tetrachloride, d-galactose and ethanol-induced liver injury (Seo et al. 2010; Zhang X et al. 2019). Flavonoid compounds were regarded as the effective substance of *C. Flos* in hepatoprotection for their satisfactory antioxidant efficacy (Zhang et al. 2000). The ethanol extract of *C. Flos* used in present study, namely BZE, is rich in flavonoids, such as apigenin, apigenin-7-*O*-β-d-glucoside and luteolin-7-*O*-β-(6″-acetyl)-glucoside. In this work, virtual prediction based network pharmacology analysis was performed to explore the hepatoprotective mechanism of BZE which was verified in APAP-induced rat model of liver injury.

Network pharmacology, as an effective tool in the mechanism exploration of complex system, is acquiring increasing attention in the basic research of TCM. Through the construction of compound-target network of *C. Flos*, we found that quercetin (**17**), kaempferol (**13**), luteolin (**15**) and acacetin (**3**) (Table 1) had higher degree among of the bioactive compounds, indicating that they might play more crucial roles in the pharmacologic action of *C. Flos*. It is worth noting that the protective activity of quercetin and luteolin has been reported in mice model of liver injury (Kwon et al. 2015; Lin et al. 2020). After target screening, GO analysis and KEGG enrichment analysis, we found the hepatoprotective mechanism of *C. Flos* might be involved in PI3K–Akt signalling pathway, negative regulation of apoptosis and inhibition of oxidative stress. In the liver injury model of rats induced by APAP, we found that BZE pre-treatment distinctly inhibited the elevation of serum ALT and AST levels, and improved liver histopathology of rat. In addition, BZE reversed the increased ROS levels in liver tissues and decreased levels of GSH and SOD in serum induced by APAP. These results indicated that the liver protective activity of BZE might be at least partly attributed to its antioxidant efficacy.

Overdose of APAP might cause the excessive generation of its toxic metabolite, *N*-acetyl-*p*-benzoquinone imine (NAPQI), which could combine with proteins to form adducts and lead to a huge surge of ROS in mitochondria (Ramachandran et al. 2018). Therefore, oxidative stress is considered as the initial event of liver toxicity caused by APAP. Nrf2, a key regulatory factor against oxidative stress, is located in the cytoplasm by binding to the negative regulator Keap1 under physiological status (Ji et al. 2015). However, oxidative stress or other stimuli could lead to the dissociation of Nrf2 from Keap1 and promote the nuclear translocation of Nrf2, inducing the expression of antioxidant genes and enzymes by binding to AREs. The antioxidant factors activated by Nrf2 include HO-1, NQO1, GSH and SOD, which are considered to be important for the body against oxidative stress (Steel et al. 2018). We observed that the expression of Nrf2 was obviously elevated in nucleus and decreased in cytoplasm of hepatocyte by BZE pre-treatment, and BZE also significantly increased the expression of HO-1 and NQO1. These data indicated that BZE could activate the Nrf2 signalling pathway, which is consistent with our previous research (Tian Z et al. 2019).

GSK3β is a multifunctional serine/threonine protein kinase involved in various cellular physiological processes and can influence the status of Nrf2. Phosphorylated GSK3β (p-GSK3β) can promote the translocation of Nrf2 to the nucleus and increase the expression of antioxidant enzymes (Chen et al. 2016). In the present study, we found that BZE promoted the phosphorylation of GSK3β in APAP-injured rat liver. Therefore, it is reasonable to speculate that BZE activates the Nrf2 signalling pathway by enhanced the phosphorylation of GSK3β, thereby inhibiting the oxidative stress caused by APAP. AMPK not only regulates cell energy metabolism, but also plays an important role in oxidative stress (Inoki et al. 2006). Activated AMPK promotes the phosphorylation of GSK3β and further regulates the oxidative stress through Nrf2 signalling pathway (Duan et al. 2017; Park et al. 2018). It is noteworthy that BZE upregulated the level of p-AMPK markedly when compared to APAP group, indicating that the antioxidant activity of BZE should be attributed to its regulation in AMPK/GSK3β/Nrf2 signalling pathway.

Network pharmacology analysis indicated that the PI3K–Akt signalling pathway was involved in the hepatoprotective mechanism of BZE. The PI3K–Akt signalling pathway plays a critical role in cell growth, cell proliferation and deregulated apoptosis (Long et al. 2021). Mitochondrial injury and oxidative stress interact with each other, thereby leading to apoptosis and necrosis of liver cells (Shi et al. 2018). In the present study, APAP administration markedly inhibited the phosphorylation of Akt, which was reversed by BZE. Significantly increased pro-apoptotic proteins, such as caspase-3 and Bax, induced by APAP were reduced by BZE treatment. BZE could promote the expression of anti-apoptotic protein Bcl-2. These results suggested that the anti-apoptotic effects of BZE might be associated with the upregulation of PI3K–Akt pathways.

The mitochondrial membrane potential is a crucial indicator of mitochondrial function, which was reduced when mitochondria are damaged. Our research presented that BZE pre-treatment increased the mitochondrial membrane potential of liver tissue in APAP-induced rats, suggesting the mitochondrial damage was mitigated by BZE. PPAR-γ and PGC-1α are key downstream targets of AMPK, taking an essential part in mitochondrial biosynthesis (Fernandes et al. 2015; Guo et al. 2018). PGC-1α can integrate and coordinate multiple transcription factors, including nuclear respiratory factor 1 (NRF1) and mitochondrial transcription factor A (TFAM). NRF1 is a transcription chaperone factor of PGC-1α, which not only regulates the transcription of nuclear genes related to the oxidative phosphorylation, but also makes effects on mitochondrial proteins encoded by mtDNA. TFAM is a nuclear-encoded protein that maintains mtDNA replication and is an important factor in mitochondrial biosynthesis (Xu et al. 2009). Our research exhibited that BZE significantly increased the levels of PPAR-γ, PGC-1α, TFAM and NRF1 in APAP-induced rat liver, indicating that BZE improved the mitochondrial function through promoting the protein expression associated to mitochondrial biosynthesis. Therefore, it is reasonable to speculate that the anti-apoptotic effect of BZE may be attributed to the improvement of mitochondrial function in APAP-induced rat liver.

## Conclusions

For the first time, the potential mechanism of BZE was investigated by network pharmacology and was partly verified by an animal experiment based on APAP-induced rats. We found that BZE could inhibit the excessive oxidative stress via GSK3β–Nrf2 pathway and reduce apoptosis through PI3K–Akt pathway. Our work provided a new perspective to understand the liver-protective mechanism of BZE, and the flower of *Bianliang ziyu* could be further developed to favourable health products in liver-protection, and not only used as an ornamental flower.

## Supplementary Material

Supplemental Material
