# Supplementary material for: Chrysanthemi Flos extract alleviated acetaminophen-induced rat liver injury via inhibiting oxidative stress and apoptosis based on network pharmacology analysis
Source: Pharm Biol. 2021 Oct 9;59(1):1376–85. doi: 10.1080/13880209.2021.1986077 (PMC8510625; doi:10.1080/13880209.2021.1986077)
Supplement: Supplemental Material [file IPHB_A_1986077_SM5128.docx]

*Supplementary materials*

***Chrysanthemi Flos* extract alleviated** **acetaminophen-induced rat liver injury via inhibiting oxidative stress and apoptosis based on network pharmacology analysis**

Yunfeng Zhou^1,3†^, Chunli Wang^1,3†^, Jiejian Kou^1,3^, Minghui Wang^1,3^, Xuli Rong^1,3^, Xiaohui Pu^1,3^, Xinmei Xie^3*^, Guang Han^1,2*^, Xiaobin Pang^1,3*^

*^1^Pharmaceutical Institute, Henan University, Kaifeng 475004, China*

*^2^Kaifeng Key Lab for Application of Local Dendranthema morifolium in Food & Drug, Kaifeng, China*

*^3^School of Pharmacy, Henan University, Kaifeng, Henan, 475004, China*

^*^**Correspondence to:**

**^*^**Xiaobin Pang, Pharmaceutical Institute, Henan University, Kaifeng 475004. Email: pxb@vip.henu.edu.cn, Tel: +86-0371-23880680

**^*^**Xinmei Xie, Pharmaceutical Institute, Henan University, Kaifeng 475004. Email: xxm@vip.henu.edu.cn

**^*^**Guang Han, Pharmaceutical Institute, Henan University, Kaifeng 475004. Email: hang@vip.henu.edu.cn

†These authors contributed equally to this work and should be considered co-first authors.

Table S1 The results of biochemical indexes in serum or liver of rats

|  |  | Control | APAP |  | APAP |  |
| --- | --- | --- | --- | --- | --- | --- |
|  |  |  |  | 110 | 220 | 440 |
| Serum | ALT (U/L) | 27.74 ± 9.54 | 66.45 ± 3.63^##^ | 40.78 ± 4.84^**^ | 24.81 ± 1.03^**^ | 22.64 ± 3.87^**^ |
|  | AST (U/L) | 21.49 ± 7.45 | 59.59 ± 4.71^##^ | 31.97 ± 4.35^**^ | 19.04 ± 0.75^**^ | 17.49 ± 2.80^**^ |
|  | SOD (U/L) | 100.67 ± 17.15 | 37.15 ± 9.9^##^ | 82.1 ± 5.49^*^ | 96.48 ± 11.56^*^ | 116.25 ± 13.74^**^ |
|  | GSH (μmol/L) | 43.33 ± 1.67 | 36.11 ± 0.96^##^ | 65.00 ± 10.23^*^ | 71.67 ± 11.67^*^ | 99.44 ± 11.88^**^ |
| Liver | DCFH-DA fluorescence intensity | 9.68 ± 0.23 | 15.19 ± 0.52^##^ | 14.01 ± 0.35^**^ | 10.7 ± 0.38^**^ | 9.95 ± 0.37^**^ |

Note: ##*p* < 0.01 *vs* Control group; **p* < 0.05; ***p* < 0.01 *vs* APAP group.
